# A *Plasmodium berghei* putative serine-threonine kinase 2 (*PBANKA_0311400*) is required for late liver stage development and timely initiation of blood stage infection

**DOI:** 10.1242/bio.042028

**Published:** 2019-08-15

**Authors:** Ravi Jillapalli, Sunil Kumar Narwal, Surendra Kumar Kolli, Babu S. Mastan, Rameswara Reddy Segireddy, Sandeep Dey, Pratik Narain Srivastava, Satish Mishra, Kota Arun Kumar

**Affiliations:** 1 Department of Animal Biology, School of Life Sciences, University of Hyderabad, Hyderabad 500046, India; 2 Division of Parasitology, CSIR-Central Drug Research Institute, Lucknow 226031, India

**Keywords:** *Plasmodium*, Serine-threonine kinases, *Uis* genes, Exo-erythrocytic forms, Hepatic schizogony, Pre-patent period, MSP1

## Abstract

In *Plasmodium*, protein kinases govern key biological processes of the parasite life cycle involved in the establishment of infection, dissemination and sexual reproduction. The rodent malaria model *Plasmodium berghei* encodes for 66 putative eukaryotic protein kinases (ePKs) as identified through modelling domain signatures and are highly conserved in *Plasmodium falciparum*. We report here the functional characterisation of a putative serine-threonine kinase *PBANKA_0311400* identified in this kinome analysis and designate it as *Pbstk2*. To elucidate its role, we knocked out *Pbstk2* locus and performed a detailed phenotypic analysis at different life cycle stages. The *Pbstk2* knockout (KO) was not compromised in asexual blood stage propagation, transmission and development in the mosquito vector. The *Pbstk2* KO produced viable salivary gland sporozoites that successfully transformed into exo-erythrocytic forms (EEFs) and were morphologically indistinguishable from wild-type GFP (WT GFP) with regard to size and shape until 48 h. An intravenous dose of 1×10^3^ *Pbstk2* KO sporozoites in C57BL/6 mice failed to establish blood stage infection and a higher dose of 5X10^3^ showed a 2–3 day delay in prepatency as compared to WT GFP parasites. Consistent with such an observation, analysis of *in vitro* EEF development at 62 h revealed that the hepatic merozoite numbers were reduced to nearly 40% as compared to WT GFP and showed meagre expression of MSP1. Our studies provide evidence for the role of *Pb*STK2 in late liver stage development and for the successful establishment of a timely blood stage infection.

## Abstract (precis)

**Summary:** Requirement of a putative serine threonine kinase 2 (PBANKA_0311400) for *Plasmodium berghei* liver stage development.

## INTRODUCTION

Malaria is a mosquito-borne infectious disease caused by a protozoan parasite that belongs to the genus *Plasmodium*. The parasite kills nearly half a million people annually with deaths predominantly occurring in sub-Saharan Africa (WHO, 2017). The parasite infects a mammalian host via the bite of a female *Anopheles* mosquito that inoculates sporozoites into the skin during a blood meal (Sinnis and Zavala, 2008). The sporozoites make their way to the liver and develop into exoerythrocytic forms (EEFs) inside hepatocytes. After several rounds of asexual reproduction, the hepatic merozoites are released into bloodstream (Prudencio et al., 2006) to initiate an erythrocytic cycle, a phase that is responsible for all clinical manifestations of malaria. Gametocytes are the terminal stages of a parasite developing within erythrocytes and do not undergo further development in the mammalian host until they arrive in the mosquito gut. Within the mosquito midgut, the parasites undergo sexual reproduction, culminating in the production of thousands of infectious sporozoites. The sporozoites migrate to salivary glands and reside there to initiate new infection cycle in the mammalian host (Matuschewski, 2006).

*Plasmodium* parasites have evolved distinct kinase families with novel domain structures and biochemical features (Ward et al., 2004). These signalling molecules play a key role in the regulation of several physiological processes (Solyakov et al., 2011). In general, phosphorylation of specific amino acid residues like serine (Ser), threonine (Thr), tyrosine (Tyr), histidine (His), and aspartate (Asp) affects the activity of target proteins either by bringing a conformational change in its active site or regulating protein–protein interactions (Pereira et al., 2011). The systematic functional investigation of *Plasmodium* *berghei* kinome by reverse genetic approach revealed that nearly two-thirds of the *P. berghei* kinases were essential (Tewari et al., 2010). While the possibility of targeting kinases essential for *Plasmodium* development in vector host may not be feasible, nonetheless several kinases seem to regulate the transmission of malaria to mosquitoes and the forms of parasite that are infective to hepatocytes can only be obtained from mosquito stage (Tewari et al., 2010). Thus it is imperative that an in-depth functional investigation of kinase mutants be done at all life cycle stages for all ‘possibly essential kinases’ such that the importance of the same kinase playing a role at multiple life cycle stages of the parasite is not overlooked and those critical for establishment of malaria infection in a mammalian host is not undermined. To date, only a few protein kinases have been identified that are required for *Plasmodium* liver stage development. The lipid kinase, phosphatidylinositol-4-OH kinase [PI(4)K] is required for hypnozoite formation in a *Plasmodium* *cynomolgi* (McNamara et al., 2013). Two mitogen-activated protein kinases (MAPKs) have also been identified in *P. berghei* and are designated as *Pb*MAPK1 and *Pb*MAPK2. While both the *Pb*MAPK genes are transcribed during *P. berghei* liver stage development, and *Pb*MAPK1 localises to the cytomere stage, depleting its locus did not affect the parasite viability in the liver stages (Wierk et al., 2013). In *P. berghei*, the cGMP-dependent protein kinase (PKG) have been implicated in liver stage schizont development, in addition to its role in ookinete differentiation and motility. The *Plasmodium* *falciparum* orthologue of PKG was shown to be required for gametogenesis and rupture of asexual blood stage schizonts (Hopp et al., 2012). Small molecule inhibitors active against liver-stage expressed kinases may offer more realistic chemotherapy as it may block the onset of clinical disease. Indeed, studies in this direction demonstrated that both genetic ablation (Falae et al., 2010) and target based drug delivery (Panchal and Bhanot, 2010) against *Plasmodium* kinases uniquely expressed in liver stages can inactivate pre-erythrocytic stages (Panchal and Bhanot, 2010; McNamara et al., 2013). For example, conditional depletion of cGMP dependent protein kinases (PKG) in sporozoite stage resulted in arresting the parasite at late liver stages that suffered from an inability to generate infectious merosomes, and mice infected with PKG mutants developed immunity that conferred protection against subsequent sporozoite challenge (Falae et al., 2010). Further PKG inhibitors effectively diminished sporozoite infectivity demonstrating the exciting feasibility of using kinase inhibitors as pre-erythrocytic antimalarials (Panchal and Bhanot, 2010). Also, a recent study demonstrated effective inhibition of *P. cynomolgi* hypnozoites by imidazopyrazines (McNamara et al., 2013).

In order to ascertain function to other kinases uniquely expressed in the pre-erythrocytic stages, we selected a putative serine-threonine kinase PBANKA_031140 for our investigation. Previous findings have shown that the *P. falciparum* orthologue of PBANKA_031140 was detected in the proteomic analysis of salivary gland sporozoites (Lasonder et al., 2008). Since salivary gland sporozoites are infective forms of the parasite to the mammalian hepatocytes, we wanted to investigate if sporozoite specific expression of PBANKA_031140 was linked to a hepatocyte infection or subsequent intrahepatic EEF development. By using a reverse genetics approach, we demonstrate the role of PBANKA_031140 in late liver stage development and initiation of a timely blood stage infection. We designated this kinase as PbSTK2 owing to the previous description a *P.* *falciparum* STK (Kuang et al., 2017).

## RESULTS

### Bioinformatic search reveals that *Pb*STK2 is conserved across rodent and human *Plasmodium* species and has calmodulin-binding motifs

**Fig. 1.**
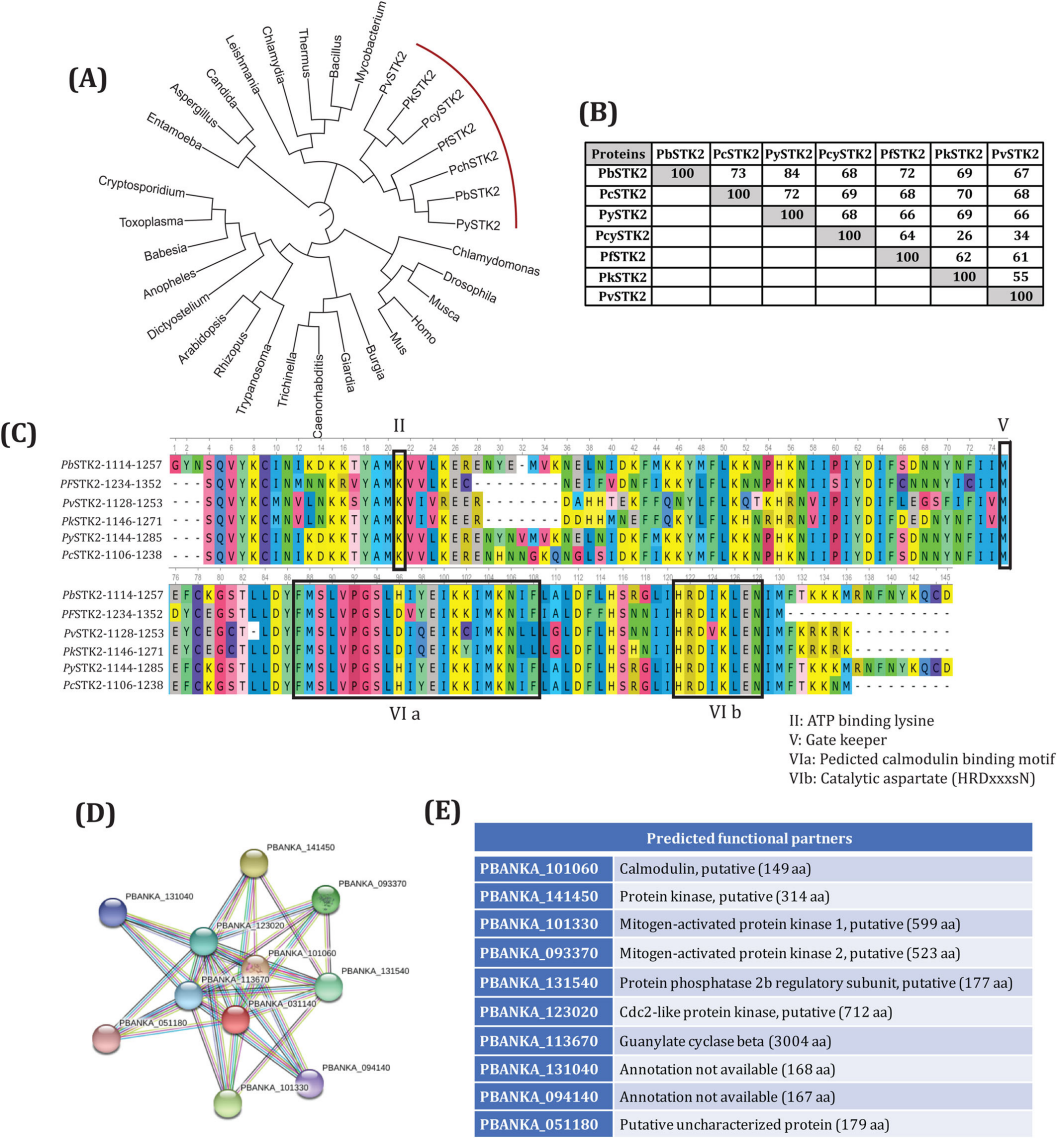
**Amino acid sequence analysis of *P. berghei* serine-threonine kinase (*Pb*STK2).** (A) Phylogenetic tree of *Plasmodium* serine-threonine kinase (STK2). *Plasmodium* STK2 orthologues present in PlasmoDB are: *P. berghei* (*Pb*STK2: PBANKA_0311400), *P. falciparum* (*Pf*STK2: PF3D7_0214600), *P. knowlesi* (*Pk*STK2: PKNH_0406200), *P. chabaudi* (*Pc*STK2: PCHAS_0313500), *P. vivax* (*Pv*STK2: PVX_002805), *P. cynomolgi* (*Pcy*STK2: PCYB_041560) and *P. yoelii* (*Py*STK2: PY06391). (B) *Plasmodium* STK2 amino acid sequence similarity matrix is represented in the table. Identities were computed using the Clustal Omega program. (C) Catalytic domain alignments *Plasmodium* STK2 orthologues. The conserved kinase domain features such as the ATP binding lysine (II), the gatekeeper methionine (V), Predicted Calmodulin binding motif (VI a) and the aspartic acid (HRDxxxsN) that acts as the catalytic residue (VI b) are conserved throughout. (D) STRING interaction diagram of *Pb*STK2. (E) The predicted STRING interaction partners of *Pb*STK2 are Calmodulin, putative (PBANKA_101060), Protein kinase, putative (PBANKA_141450), Mitogen-activated protein kinase 1 (PBANKA_101330), Mitogen-activated protein kinase 2 (PBANKA_093370), Protein phosphatase 2b regulatory subunit, putative (PBANKA_131540), Cdc2-like protein kinase, putative (PBANKA_123020), Guanylate cyclase beta (PBANKA_113670), centrin-2, putative (PBANKA_131040), centrin-4, putative (PBANKA_094140), centrin-3, putative (PBANKA_051180).

Phylogenetic analysis revealed *Pb*STK2 is conserved among the Plasmodial species but is not related to any other organisms used for the analysis (Fig. 1A). Alignment of *Pb*STK2 (PBANKA_0311400) amino acid sequence from various rodent and human Plasmodial species revealed the presence of highly conserved orthologues (Fig. 1B). The maximal degree of conservation noted was 84% with *Plasmodium* *yoelii* STK2 (*Py*STK2, PY17X_0311900) followed by 73% with *Plasmodium* *chabaudi* (*Pc*STK2, PCHAS_0313500), 72% with *P. falciparum* (*Pf*STK2, PF3D7_0214600), 69% with *Plasmodium* *knowlesi* (*Pk*STK2, PKNH_0406200), 68% with *P. cynomolgi* (*Pc*STK2, PCYB_041560) and 67% with *Plasmodium* *vivax* (*Pv*STK2, PVX_002805) (Fig. 1B). Multiple sequence alignment (MSA) of the kinase domain of *Pb*STK2 shown in Fig. 1C revealed a high degree of amino acid conservation across all *Plasmodium* orthologues.

The Ser/Thr protein kinases interact with diverse substrates like enzymes, other kinases, transcription factors, receptors and regulatory proteins (Goldsmith et al., 2007). Fig. 1D depicts the STRING interaction network of *Pb*STK and Fig. 1E details its possible interacting partners based on co-expression of genes. These included several putative candidates like calmodulin (PBANKA_101060), a protein kinase (PBANKA_141450), mitogen-activated protein kinase 1 (PBANKA_101330), mitogen-activated protein kinase 2 (PBNAKA_093370), a protein phosphatase 2b regulatory subunit, (PBANKA_131540), a Cdc2-like protein kinase (PBANKA_123020), guanylate cyclase beta (PBANKA_113670), centrin-2, putative (PBANKA_131040), centrin-3, putative (PBANKA_051180) and centrin-4, putative (PBANKA_094140). The STRING prediction of *Pb*STK2 interaction with calmodulin is consistent with its inclusion under the CaMK group (Tewari et al., 2010). Calmodulin binding motifs type 1–10 and 1–16 are found in CaM-dependent PKs and CaM-dependent KKs, respectively. From sequence analysis and local similarity search, both motif types were found by matching the calmodulin binding peptide sequence obtained from PDB structures of calmodulin complexes with the *Pb*STK2 amino acid sequence. Motif type 1–10, similar to calmodulin binding peptide of Human CaMKII (PDB ID: 3GP2), occurred in *Pb*STK2 at 1505-1511 (SFKKRRK) and motif type 1–16, similar to calmodulin binding peptide of Rat CDPK (PDB ID: ICKK), occurred in *Pb*STK2 at 1200-1217 (FMSLVPGSLHIYEIKKIMKNIF) (Fig. 1C). The prediction of *Pb*STK2 1200-1217 as calmodulin-binding motifs is more reliable as the site contains two phenylalanine (F) residues separated by 20 residues. The secondary structure prediction of this peptide reveals it to be mostly helical, a requirement for calmodulin binding. The prediction of *Pb*STK2 1505-1511 (1–10) is not very reliable as it does not contain the conserved F residues at proper positions (Osawa et al., 1999).

### *Pbstk2* is maximally expressed in salivary gland sporozoite stage

**Fig. 2.**
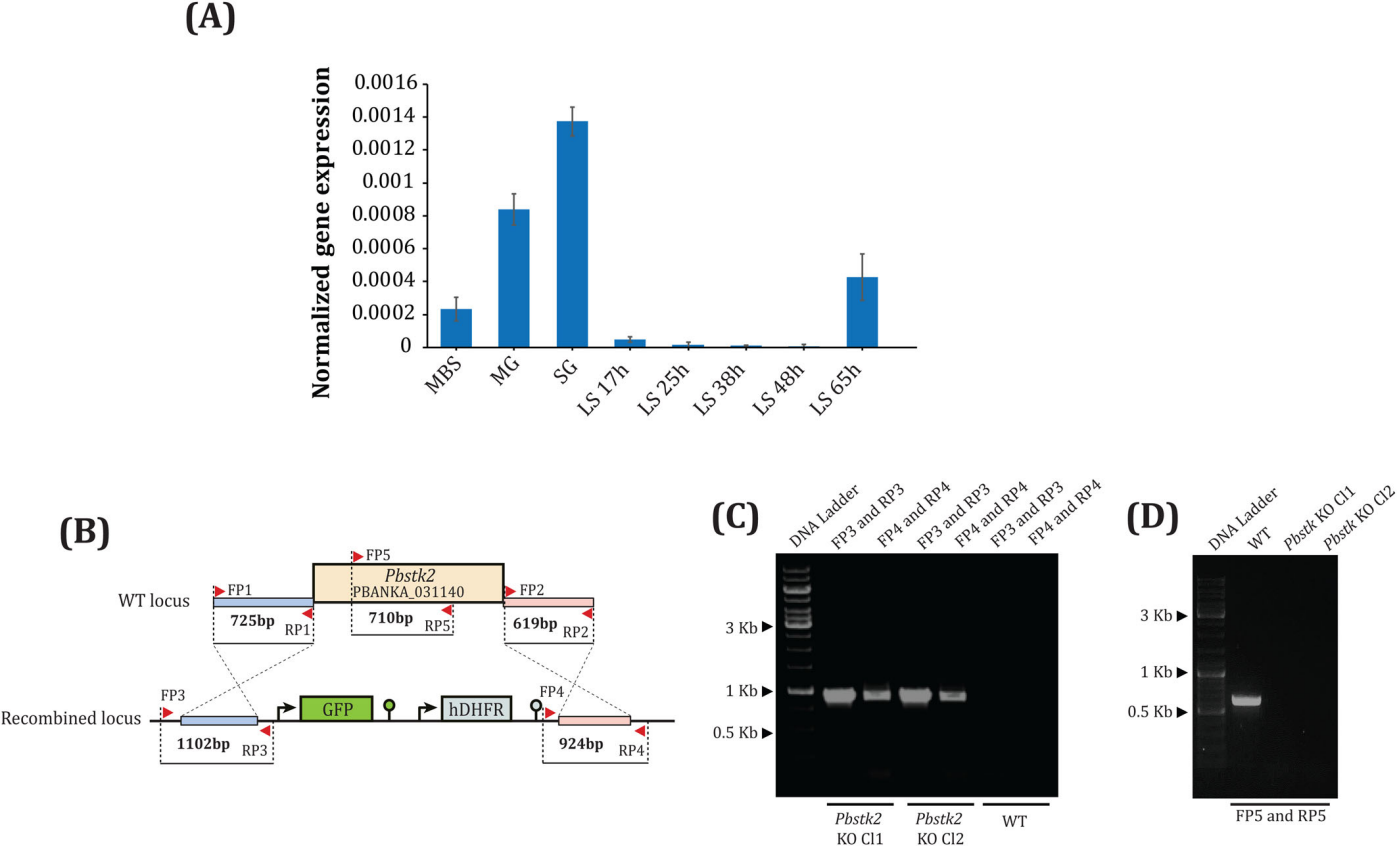
***Pbstk2* gene expression analysis and generation of *Pbstk2* knockout (KO).** (A) Stage-specific expression of *Pbstk2* by quantitative real-time PCR (qRT-PCR). The samples included, mixed blood stages (MBS), day 14 midgut oocyst stage (MG), day 18 salivary gland sporozoite stage (SG), *in vitro* grown liver stages (LS) in hours (h) at 17, 25, 38, 48 and 65. Values were normalized to *P. berghei 18s rRNA*. (B) Strategy for targeting the *Pbstk2* (PBANKA_031140) locus by double cross-over homologous recombination. A 725 bp and 619 bp of 5′ and 3′ region of *Pbstk2* was amplified using primers FP1-RP1 and FP2-RP2, respectively. Both fragments were cloned into a pBC-GFP-hDHFR vector using *Xho*I-*Cla*I for the 5′ region and *Not*I-*Asc*I for the 3′ region. (C) Diagnostic PCR showing the correct integration of targeting construct at *Pbstk2* locus in Cl1 and Cl2 KO lines. The correct integration of the 5′ region was confirmed by a product of 1102 bp amplified using primers FP3 and RP3. The correct integration of the 3′ region was confirmed by a product of 924 bp amplified using primers FP4 and RP4. No product was amplified from genomic DNA of WT parasites using the aforementioned primer sets. (D) Amplification of a 710 bp fragment within *Pbstk2* ORF using primer sets FP5 and RP5 from WT parasite genomic DNA but not from Cl1 and Cl2 confirmed successful establishment of Cl1 and Cl2 lines.

Stage-specific gene expression of *Pbstk2* was analysed by standard quantitative real-time PCR (qRT-PCR) using cDNA samples generated from mixed blood stages, midgut sporozoites, salivary gland sporozoites and liver stages at 17 h, 25 h, 38 h, 48 h and 65 h. We noted maximal expression in salivary gland sporozoite stage followed by midgut sporozoite stage and at 65 h liver stages (Fig. 2A).

### *Pbstk2* locus is not refractory to genetic manipulation

The strategy for the generation of *Pbstk2* KO is shown in Fig. 2B. The 5′ fragment and 3′ fragment of *Pbstk2* were amplified using primer sets FP1-RP1 and FP2-RP2 that yielded, respectively, products of 725 bp and 619 bp. The stable integration of the pBC-GFP-hDHFR carrying the 5′ and 3′ region of *Pbstk2* at the desired locus was confirmed by diagnostic PCR. The primer sets FP3-RP3 and FP4-RP4 amplified products of 1102 bp and 924 bp, respectively, from both *Pbstk2* KO clones Cl1 and Cl2, but not from WT GFP indicating the correct genomic integration of the *Pbstk2* KO construct (Fig. 2C). Additionally, we also sequenced the *Pbstk2* KO locus and confirmed the presence of GFP-hDHFR cassette (Fig. S1). Further, a primer set FP5-RP5 designed within *Pbstk2* ORF amplified a product of 710 bp from WT GFP line but not from Cl1 and Cl2 (Fig. 2D).

### *Pbstk2* KO parasites develop normally during asexual blood stages and in mosquito stages

**Fig. 3.**
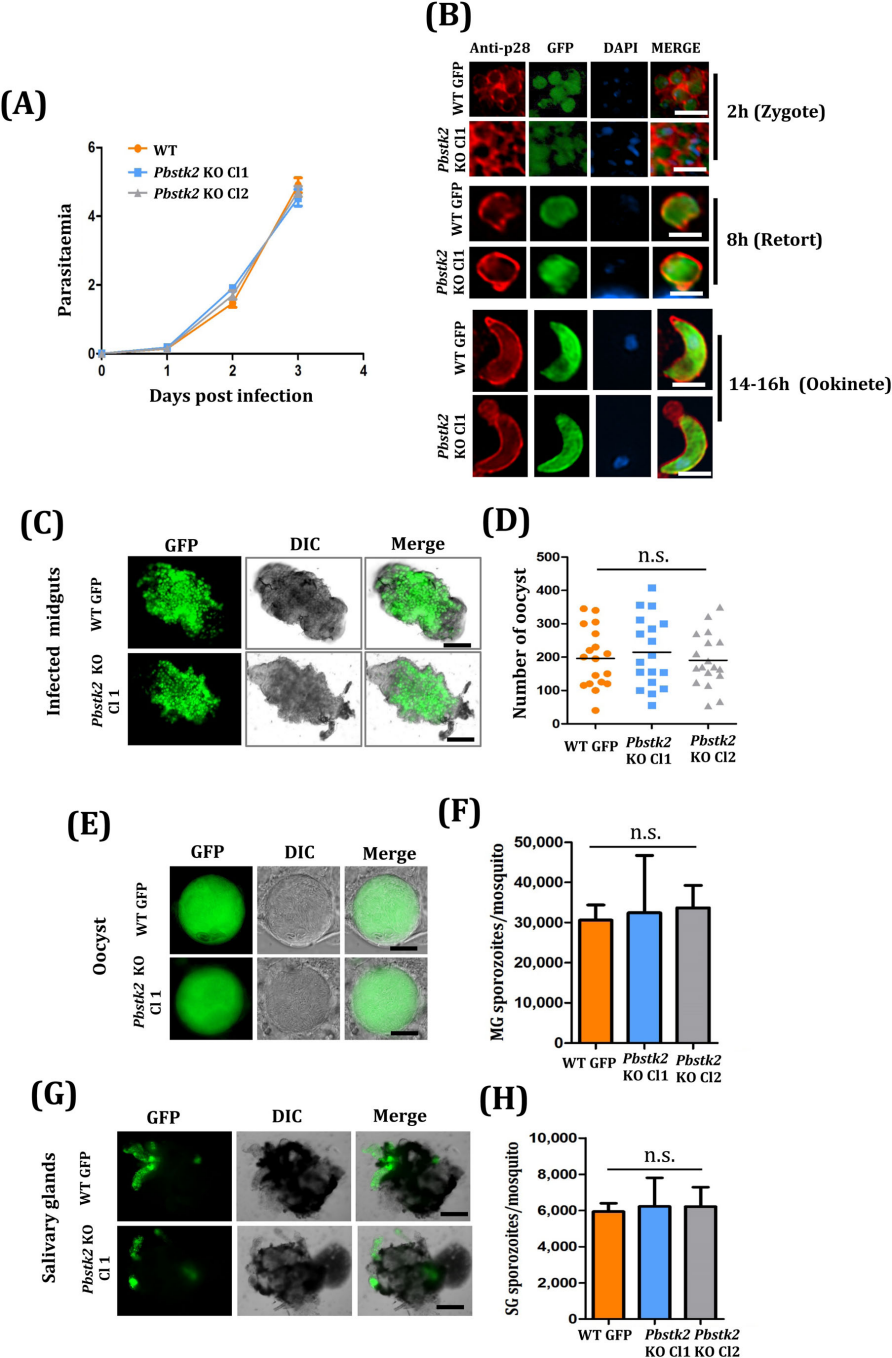
**Phenotypic characterisation of *Pbstk2* KO in asexual and mosquito stages.** (A) Asexual propagation of WT GFP and *Pbstk2* KO Cl1 and Cl2 lines in mice. (B) Anti-p28 staining of the zygote (2 h), retort stage (8 h) and ookinete stage (20 h) in WT GFP and *Pbstk2* KO clone Cl1. Scale bars: 5 µm. (C) Midguts showing oocyst in WT GFP and *Pbstk2* KO cl1 line. Scale bars: 200 µm. (D) Quantification of oocysts in WT GFP and *Pbstk2* KO clones Cl1 and Cl2. (E) Sporulation within oocyst in WT GFP and *Pbstk2* KO cl1. Scale bars: 20 µm. (F) Quantification of oocyst sporozoites in WT GFP and *Pbstk2* KO clones Cl1 and Cl2. (G) Mosquito salivary glands showing WT GFP and *Pbstk2* KO cl1 sporozoites. Scale bars: 200 µm. (H) Quantification of salivary gland sporozoites in WT GFP and *Pbstk2* KO clones Cl1 and Cl2.

With the cloned lines of *Pbstk2* KO, we analysed the asexual blood stage propagation as compared to WT GFP. Two groups of Swiss mice received intraperitoneal injection of 200 µl infected blood (having 0.2% parasitemia) from both cloned lines and WT GFP parasites. Parasitemia was monitored daily, by making Giemsa stained blood smears. We observed no difference in the blood stage propagation of *Pbstk2* KO parasites as compared to WT GFP thus confirming the dispensable role of *Pb*STK2 in asexual blood stage propagation (Fig. 3A).

We next investigated the role of *Pbstk2* KO in sexual stage development. *In vitro* cultures were set up from gametocyte positive blood of *Pbstk2* KO Cl1, Cl2 and WT GFP. The cultures were harvested at 2 h, 8 h and 20 h and stained with anti-p28 antibody, a marker for zygote, retort and ookinete stage. We observed normal development of both *Pbstk2* KO clones (data is shown for *Pbstk2* KO Cl1) during sexual stages that were comparable to WT GFP (Fig. 3B). The midgut infectivity [Fig. 3C,D (data shown for *Pbstk2* KO Cl1)], sporulation within oocyst [Fig. 3E, (data shown for *Pbstk2* KO Cl1)], oocyst sporozoite loads (Fig. 3F) and salivary gland sporozoite loads [Fig. 3G,H (data shown for *Pbstk2* KO Cl1)] in both *Pbstk2* KO clonal lines were comparable to WT GFP parasites. We conclude that PbSTK2 has no role in *Plasmodium* development that occurs within the mosquito vector.

### *Pbstk2* KO sporozoites exhibit normal gliding motility, cell traversal activity, and infectivity

**Fig. 4.**
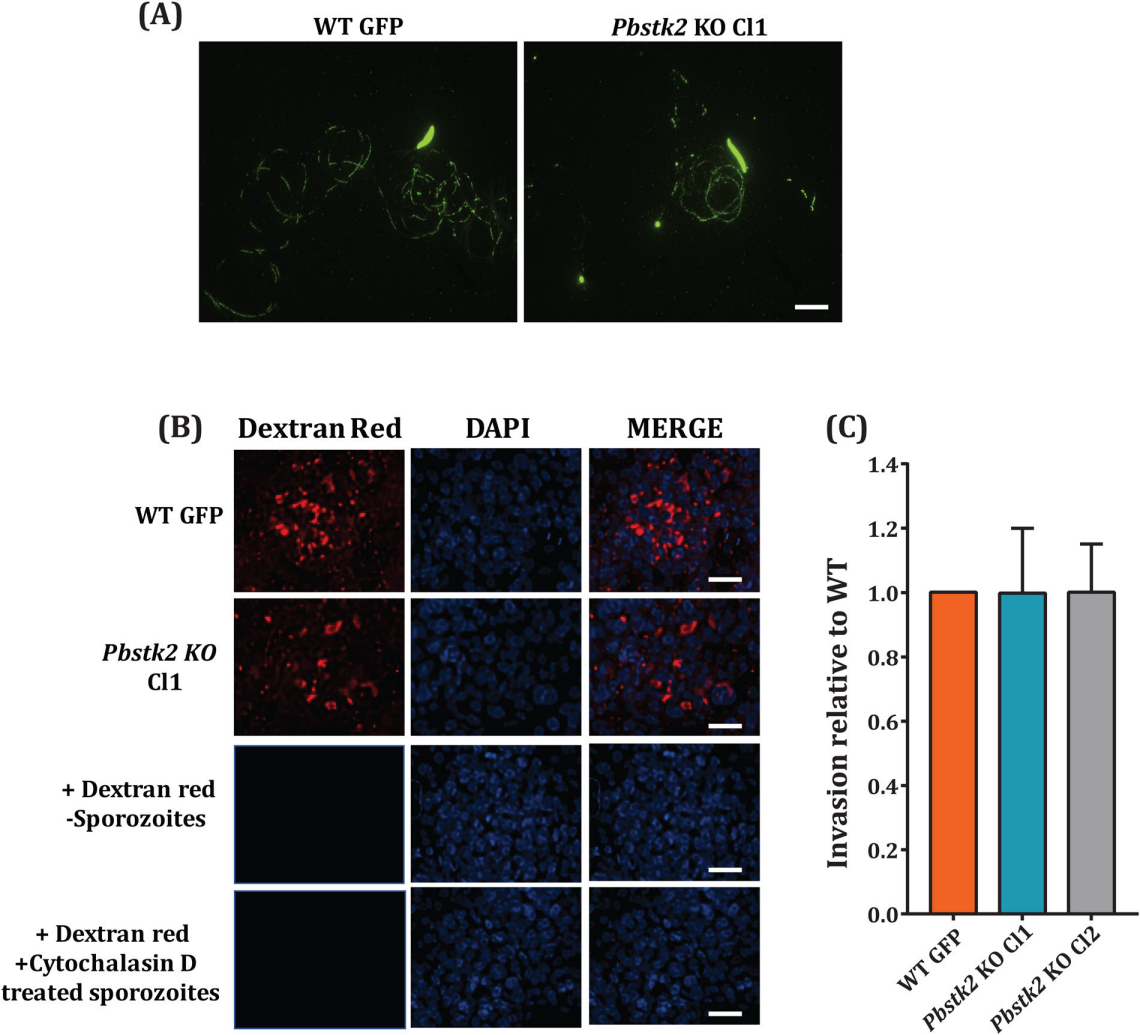
**Characterisation of *Pbstk2* KO sporozoite gliding motility and *in vitro* infectivity.** (A) Gliding motility of WT GFP and *Pbstk2* KO Cl1 sporozoites. Scale bar: 10 μm. (B) Cell traversal assay for WT GFP and *Pbstk2* KO Cl1 sporozoites in the presence of rhodamine-labelled dextran. The specificity of rhodamine-labelled dextran accumulation inside cells was evident by lack of labelling in the absence of sporozoites or under conditions of treating sporozoites with cytochalasin D. (C) Quantification of intracellular infectivity of *Pbstk2* KO by sporozoite inside out assay. Equal numbers (2×10^4^) sporozoites of WT GFP and *Pbstk2* KO clones Cl1 and Cl2 were added to monolayers of HepG2 cells. One hour later, the cultures were fixed and the extracellular sporozoites were stained with anti-CSP monoclonal antibody 3D11 and the immunoreactivity was revealed using anti-mouse secondary antibody conjugated to Alexa Fluor. The slides were observed under fluorescent microscope Ni-AR and in each field, the total number of sporozoites (GFP positive) and a number of extracellular sporozoites (red fluorescence) were counted and the invasion efficiency related to WT GFP was calculated. Scale bar: 20 μm.

Next, we studied activities of *Pbstk2* KO sporozoite required for hepatocyte infection. Sporozoites rely on actin-based gliding motility that facilitates host cell invasion (Kappe et al., 2004) and crossing of cellular barriers *in vivo*. The gliding pattern of *Pbstk2* KO sporozoite was similar to WT GFP (Fig. 4A, data is shown for *Pbstk2* KO Cl1). We next tested the cell traversal activity of sporozoite. The *Pbstk2* KO were able to traverse through monolayers of HepG2 cells as visualised by deposition of rhodamine-labelled dextran (data is shown for *Pbstk* KO Cl1) as noted for WT GFP. Lack of labelling in HepG2 cultures in the absence of sporozoites or following addition cytochalasin D treated sporozoites (inhibitor of actin polymerisation) confirmed the specificity of labelling (Fig. 4B). Quantification of *Pbstk2* KO sporozoite infectivity in HepG2 cells by inside out assay revealed no apparent defect in both *Pbstk2* KO clones in colonising hepatocytes (Fig. 4C).

### *Pbstk2* KO manifest a delay in the pre-patent period

To analyse the ability of *Pbstk2* KO sporozoites to induce infection in a mammalian host, we intravenously infected C57BL/6 mouse with two different doses viz., 5×10^3^ or 1×10^3^ of WT GFP and *Pbstk2* KO sporozoites and analysed the pre-patent period. At 5×10^3^ dose, the *Pbstk2* KO sporozoites became patent on day 5–7 as compared to day 3 for WT GFP. A dose of 1×10^3^ *Pbstk2* KO sporozoites was unable to initiate blood stage infection as judged by the complete absence of pre-patent period till day 30 (Table 2). We conclude that low doses of *Pbstk* KO sporozoites fail to cause infection while high doses cause occasional breakthrough infection.

### *Pbstk* KO late liver stages show reduced hepatic schizogony and MSP1 expression

**Fig. 5.**
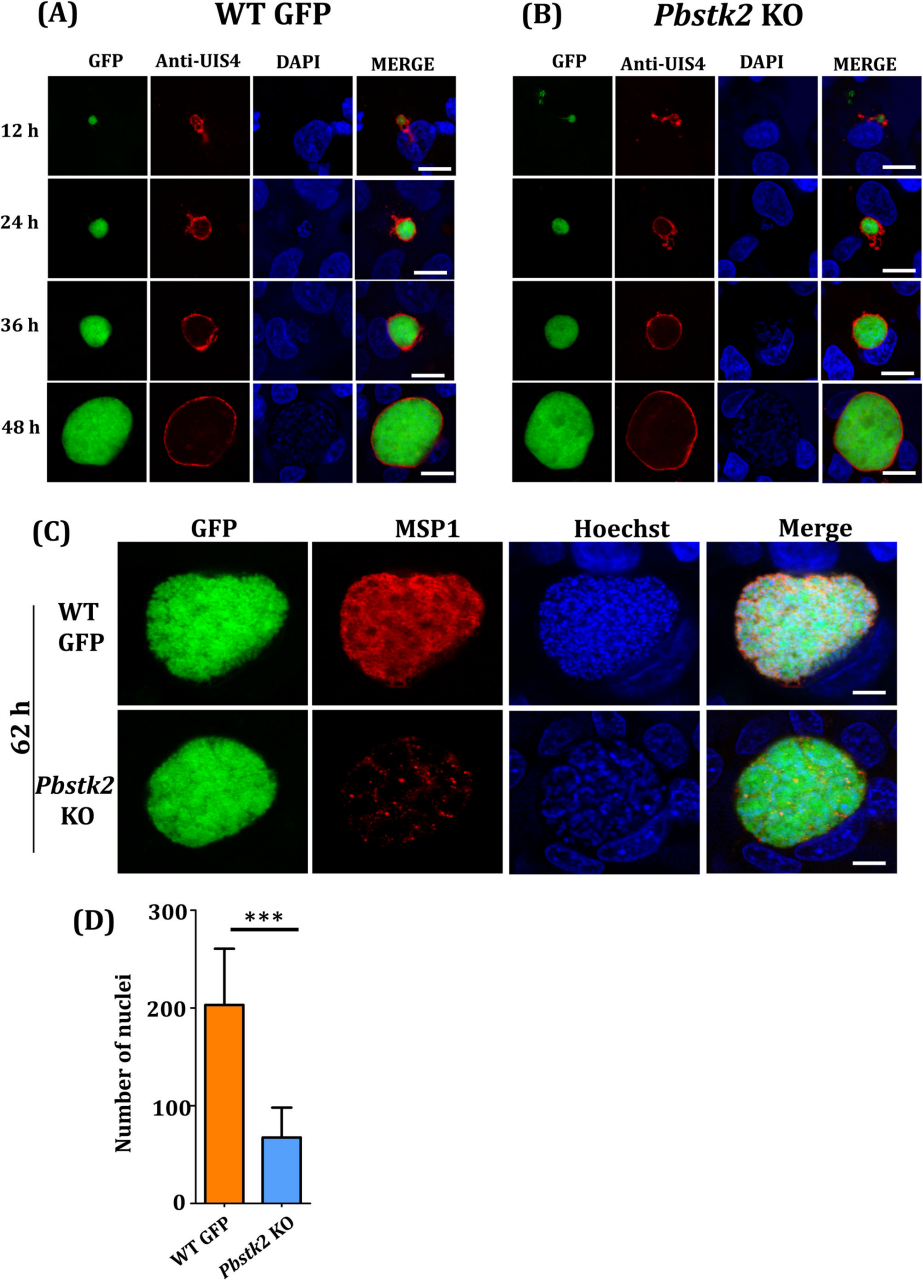
***In vitro* EEF development of *Pbstk2* KO.** The *in vitro* EEF development of (A) WT GFP and (B) *Pbstk2* KO Cl1 at different time points in hours (h) as indicated. The parasitophorous vacuole was stained with anti-UIS4 antibody and the immunoreactivity was revealed using anti-rabbit secondary antibody conjugated to Alexa Fluor 594. The parasite and host nuclei were stained with Hoechst 33342. Scale bars: 10 µm. (C) *In vitro* EEF development at 62 h revealed significantly reduced MSP1 expression in *Pbstk2* KO Cl1. The MSP1 staining was revealed using anti-mouse secondary antibody conjugated to Alexa Fluor 594. The parasite and host nuclei were stained with Hoechst 33342. Scale bars: 10 µm. (D) Quantification of hepatic merozoite nuclei at 62 h (***P<0.0001, Student’s *t*-test).

A delay in the pre-patent period implicated an obvious growth defect in EEF development in the *Pbstk2* KO. In order to identify precise time point of the defect, *Pbstk2* KO sporozoites were added to monolayers of HepG2 cultures and their development was monitored at various time points by staining with anti-UIS4 antibody, a marker for parasitophorous vacuolar membrane (PVM). HepG2 cell and parasite nuclei were stained with Hoechst 33342. We observed no difference in the growth of EEFs at all time points tested in WT GFP (Fig. 5A) and *Pbstk2* KO clone 1 (Fig. 5B).

To account for the delay in prepatency of *Pbstk2* KO, we analysed 62 h liver stage culture by staining with monoclonal antibody 25.1 specific for merozoite surface protein 1 (MSP1). Hoechst 33342 was used for nuclear staining. We noted dramatic decrease in MSP1 expression in *Pbstk2* KO EEFs (Fig. 5C) as compared to WT GFP. The nuclei of hepatic merozoites were quantified using ImageJ software (https://imagej.nih.gov/ij). We noted that the hepatic merozoite numbers were reduced to nearly 40% as compared to WT GFP (Fig. 5D). We conclude that loss of *Pb*STK2 expression led to significant reduction in schizogony that affected both hepatic merozoite maturation and MSP1 expression thus preventing or delaying blood stage infection.

## DISCUSSION

Identifying protein kinases that play a role during pre-erythrocytic stages of *Plasmodium* life cycle holds the potential to develop inhibitors that may delay or prevent blood stage infection. In this direction, we prioritised investigating the role of *Pb*STK2 owing to its detection in the proteomic analysis of salivary gland sporozoites (Lasonder et al., 2008). Consistent with the high levels of *Pbstk2* transcripts and occurrence of protein in salivary gland sporozoites, our study demonstrates its requirement at 65 h liver stage for successful completion of hepatic schizogony and initiation of blood stage infection.

Bioinformatic analysis predicted one or more calmodulin-binding domains in *Pb*STK2 suggesting that it belongs to the family of CaMK and concurs with previous studies (Tewari et al., 2010). CaMKs are activated by an increase in intracellular concentration of Ca^2+^ ions leading to the transfer of phosphates from ATP to defined serine or threonine residues of the substrate proteins. In view of our observation that *Pb*STK2 is required to complete hepatic schizogony, it is likely possible that this kinase may be activated by intracellular Ca^2+^ that raises transiently in hepatic merozoites to prevent apoptosis (Sturm et al., 2006). This may explain partially as to why the mutants that lack *Pb*STK2 activity are impeded in completion of liver stage development. The fact that *Pbstk2* KO showed an overall reduced hepatic schizogony may also point to its likely role in the regulation of the cell cycle, given that CaMKs influence the activity of cell division cycle (CDC) regulators (Sur and Agrawal, 2016). Further investigations are required to ascertain such hypotheses.

CaMKs have also been shown to play a role in other life cycle stages. In *Plasmodium gallinaceum* morphologic differential from zygote to ookinete stage was shown to be dependent on CaMK. Cellular extracts of zygote and ookinete phosphorylated autocamtide-2, a classic CaMK substrate that was blocked by both calmodulin antagonists W-7 and CaMK inhibitor KN-93 (Silva-Neto et al., 2002). While this study did not precisely identify the parasite-specific CaM kinase that was sensitive to the aforementioned inhibition, the fact that *Pbstk2* mutants did not manifest any phenotype in the zygote and ookinete stage implied the presence of multiple CaM kinases that may have stage-specific functions.

Of the 66 *P. berghei* ePKs characterised previously (Tewari et al., 2010), 43 kinases were refractory to genetic manipulation and *Pbstk2* was inclusive in this group. More recent studies of Bushell et al. (Bushell et al., 2017) also report this candidate to likely be essential for blood stages. However, we were able to successfully disrupt *Pbstk2* locus. A likely explanation for this discrepancy, as reiterated previously (Tewari et al., 2010), may be the limitations of working with a rodent model, where gene targeting may not be effective every time and hence the inability to disrupt a gene does not necessarily point to its essential nature. However, piggyback screen performed in *P. falciparum* indicated that *stk2* (PF3D7_0214600) is dispensable in the blood stages (Zhang et al., 2018). The other kinases classified as ‘possibly essential’ therefore merits a thorough functional investigation in order to unravel their unique stage-specific functions.

Our studies provide a possibility of targeting *Pb*STK2 to prevent blood stage infections for two important reasons. Firstly, because of its conserved nature across all *Plasmodium* species and its distinctness from the human counterpart, a *Pb*STK2 inhibitor may likely block the parasite's ability to cause break through infection. Secondly, owing to its requirement for late liver stage development, *Pbstk2* locus can be an additional target while considering a multiple attenuated mutant solely based on late liver stage genes. Such mutants may have the dual advantage of conferring cross-stage immunity as well as reducing the risk of breakthrough infection. Taken together, our studies provide evidence for the first time of a role for *Pb*STK2 in late liver stage development that may have important therapeutic implications for preventing clinical malaria.

## MATERIALS AND METHODS

### Ethics statement

All animal experiments performed in this study were approved by the Institutional Animal Ethics Committee at CSIR-Central Drug Research Institute, India (approval no: IAEC/2013/83) and Institutional Animal Ethics Committee at University of Hyderabad (approval no: UH/SLS/IAEC/2014-1/9b and UH/SLS/IAEC/2014-1/9c).

### *In silico* analysis of *P. berghei* STK2

The amino sequence of *P. berghei* STK2 (PBANKA_031140) was downloaded from PlasmoDB (www.plasmodb.org), and phylogenetic data was obtained using BLAST and analysed for its closest orthologues in various groups such as bacteria, plants, fungi, animals and apicomplexans. The amino acid sequences of all the orthologues were aligned and constructed into a phylogenetic tree by ClustalX (Larkin et al., 2007). The tree was visualized and illustrated by the Interactive Tree of Life web service (Letunic and Bork, 2016). Multiple sequence alignment (MSA) of the kinase domain of *Pb*STK was performed with other species of *Plasmodium* to study its residue conservation. ClustalX was used to create the alignment, and it was visualized and illustrated by Unipro UGENE (Okonechnikov et al., 2012). STRING interactions database was used to draw an interaction network for *Pb*STK (Szklarczyk et al., 2015). The network does not necessarily point out direct protein–protein interactions but it indicates the relation of a protein with others by co-expression, co-evolution, direct interaction evidence, etc. This network helps to study the function of the protein in a broader genomic context.

### Quantitative gene expression analysis of *Pbstk2* across all the life cycle stages of *P. berghei*

The expression of the *Pbstk2* was measured by quantitative real-time using absolute quantification method. RNA was isolated from mixed blood stages, midgut sporozoites, salivary gland sporozoites and *in vitro* liver stages, at time points 17 h, 25 h, 38 h, 48 h and 65 h post-infection using Trizol (Invitrogen), and purified using RNA isolation kit (Life Technologies) according to the manufacturer's instructions. Nearly 2 µg of RNA from aforementioned stages were used for reverse transcription in a reaction mixture containing 1× PCR buffer, 2.5 mM dNTPs, 5 mM MgCl2, 1.5 units RNase inhibitor, 2.5 mM random hexamers and 1.5 units reverse transcriptase (Applied Biosystems). Gene-specific standards were generated for *Pbstk2* by amplifying 120 bp product from *P. berghei* genomic DNA using forward primer-*Pbstk2* TA FP and reverse primer *Pbstk2* TA RP and ligated into pTZ57R/T vector. *Pb18S rRNA* standard was used as an internal control (Kumar et al., 2004). The *Pbstk2* plasmid standards were generated in a log fashion ranging from 10^8^ to 10^2^ copies per μl for both *Pbstk2* and *Pb18S rRNA*. The cDNA samples were run alongside with both standards. Quantitative real-time PCR (qRTPCR) was carried out in a 10 μl reaction containing SYBR Green PCR Master Mix (Bio-Rad) and 0.25 μm gene-specific primers. qRTPCR was performed using the EFFENDORF REALPLEX 2 qPCR machine. A ratio of transcript numbers of *Pbstk2* and *Pb18S rRNA* was obtained to normalize the gene expression data (Vaughan et al., 2009).

### Generation of *Pbstk2* (PBANKA_031140) KO construct

Double homologous recombination strategy was used to delete *Pbstk2* locus. To achieve this, *Pbstk2* KO targeting construct was generated using pBC-GFP-hDHFR vector. *Plasmodium* *berghei* genomic DNA was used as a template to amplify 725 bp of 5′ upstream region of *Pbstk2* using *Pbstk2* 5′ forward primer-FP1 and *Pbstk2* 5′ reverse primer-RP1. A 619 bp of 3′ downstream region of *Pbstk2* was also amplified using *Pbstk2* 3′ forward primer-FP2 and *Pbstk2* 3′ reverse primer-RP2. PCR was set up with 1× PCR buffer, 0.25 µm FP, 0.25 µm RP, 1 mM dNTPs each (Thermo Fisher Scientific, Cat No. R72501), 2.5 mM MgCl_2_, 30–50 ng genomic DNA and 2.5 units of Taq DNA polymerase (Thermo Fisher Scientific, Cat No. 11615010). Thermal cycling was performed in Eppendorf Mastercycler PCR machine, and the cycling conditions were as follows: initial denaturation at 94°C for 2 min and 94°C for 30 s, annealing at 54°C 30 s followed by synthesis at 72°C for 1 min. The cycles were repeated 35 times except for initial denaturation and final extension was done at 72°C for 10 min. The PCR product corresponding to 5′ and 3′ regions were confirmed by sequencing. The 5′ and 3′ PCR amplified products were cloned into the pBC-GFP-hDHFR vector at *Xho*I/*Cla*I and *No*tI/*Asc*I sites, respectively. The knockout plasmid was digested with *Xho*I*/Asc*I and gel purified and its concentration was determined using NanoDrop 2000.

### *Plasmodium berghei* transfection and confirmation of stable integration of targeting construct at *Pbstk2* locus

Electroporation of the targeting construct was done essentially as described previously (Janse et al., 2006). In brief, blood was collected from mice having 4–5% parasitemia and an overnight culture was set up in RMPI medium containing 20% FBS (Gibco, South American origin) and 0.35 mg/ml gentamycin (Gibco). The next day the cultures were subjected to density gradient centrifugation using 70% nycodenz (Sigma-Aldrich). The purified schizonts were used for electroporation of the *Pbstk2* KO construct using Amaxa nucleofector device, program U033 (Janse et al., 2006). Two independent transfections were performed, and the schizonts were immediately injected intravenously into Swiss mice. The following day, blood smears were made from mouse harbouring transfected parasites and, after confirming parasitemia, the mice were subjected to an antimalarial pyrimethamine that was administered orally through drinking water. After 7 days of stringent drug selection, when the parasitemia reached around 5%, genomic DNA was isolated using a genomic DNA isolation kit (Genetix), following the manufacturer's instructions. To confirm the site-specific integration, diagnostic primers were designed within the targeting construct and beyond the site of integration at both the 5′ and 3′ ends of recombination using primer sets FP3, RP3 and FP4, RP4. Limiting dilution was done to isolate single clonal population and to eliminate non transfectants. Single clones were further confirmed for the absence of wild-type contamination by diagnostic PCR using a forward primer-FP5 and reverse primer-RP5 within the *Pbstk2* ORF. The information of all primers used in this study is indicated in Table 1.

**Table 1.**
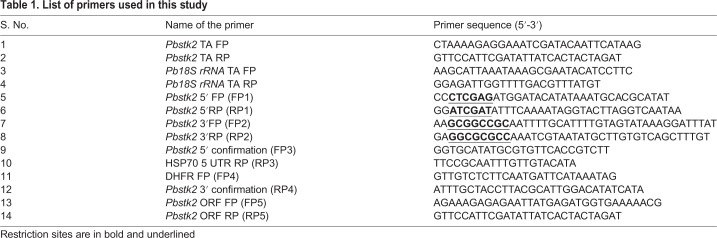
**List of primers used in this study**

**Table 2.**
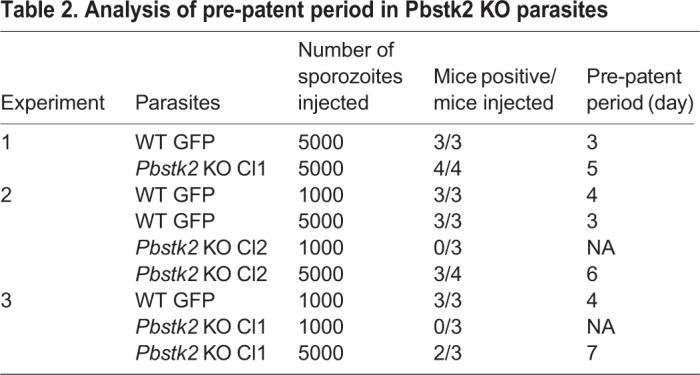
**Analysis of pre-patent period in Pbstk2 KO parasites**

### *In vitro* ookinete culture

Ookinetes were enriched by a protocol as described previously (Sinden et al., 1985) with slight modification. Swiss albino mice were treated with 1.2 mg phenylhydrazine (Sigma-Aldrich), in 0.9% NaCl, 2–3 days prior to infection. WT GFP (Al-Nihmi et al., 2017) or *Pbstk2* KO parasites were intraperitoneally injected into phenylhydrazine treated mice. When parasitemia reached to 8–10%, 30 µl of blood was collected from the tail vein. Infected blood was added to 2 ml RPMI 1640 supplemented with 24 mM sodium bicarbonate, 50.000 I.U. neomycin (Stock solution of 10.000 µg/ml; Gibco) and 20% (v/v) FBS (Gibco). These were samples were incubated at 20°C. Zygote, retorts and ookinetes were harvested at 2 h, 8 h, and 20 h, respectively.

### Transmission of Pbstk2 KO to *Anopheles stephensi* mosquito

*Anopheles* *stephensi* mosquitoes were fed on anesthetized Swiss mice blood carrying gametocytes from either WT GFP or *Pbstk* KO clones. Infected mosquitoes were kept at 19°C and 80% humidity under a 12/12 h light/dark cycle. Transmission of parasites to mosquitoes was monitored by observing the oocysts in mosquito midgut on day 14 post-blood meal. Mosquitoes were dissected to obtain salivary glands, which were then gently disrupted and debris was removed by centrifugation. Sporozoites were counted in a hemocytometer.

### Gliding motility

Gliding motility assays were performed as described previously (Stewart and Vanderberg, 1988). Briefly, Lab-Tek wells were coated overnight with 10 µg/ml of mAb 3D11 diluted in PBS and kept at 25°C. WT GFP or *Pbstk2* KO salivary gland sporozoites were dissected and added to Lab-Tek wells and incubated for 1 h at 37°C. The sample was subsequently fixed in 4% PFA and stained with biotinylated mAb 3D11 followed by streptavidin-FITC (Invitrogen) to visualise the CS protein-containing trails.

### Sporozoite cell traversal assay

To examine the cell traversal activity, WT GFP or *Pbstk2* KO sporozoites were added in Lab-Tek wells seeded with HepG2 cells. The HepG2 cells were procured from NCCS, Pune, India and were confirmed negative for mycoplasma contamination. The sporozoite invasion was performed in the presence of 1 mg/ml rhodamine-labelled dextran (10,000 MW, neutral; Invitrogen). Cells traversed by sporozoites captures dye-labeled dextran. Dextran-positive cells were enumerated manually under the fluorescent microscope (Mota et al., 2001).

### Sporozoite invasion assay

HepG2 cells were plated in eight-chambered Lab-Tek wells and were maintained in DMEM (Gibco, supplemented with L-glutamine and glucose) medium containing 10% FBS (Gibco) and allowed to grow until they reached subconfluency. Nearly 2×10^4^ sporozoites of WT GFP or *Pbstk2* KO were added in each well and following 1 h incubation at 37°C, the cells were washed and fixed with 4% paraformaldehyde. Extracellular sporozoites were in stained without permeabilising the cultures using 3D11 monoclonal antibody specific for the repeat region of CSP (Yoshida et al., 1980) and the immunoreactivity was revealed using anti-mouse secondary antibody conjugated to Alexa Fluor 594. To enumerate the sporozoite infectivity, the number of GFP expressing sporozoites (total) were counted per field and the number of sporozoites positive for CSP expression (extracellular sporozoites) was enumerated per field (Renia et al., 1988). The percentage sporozoite infection was calculated by using the formula: the number of GFP expressing sporozoites per field - number of sporozoites stained red per field/number of GFP expressing sporozoites per field × 100.

### Determination of pre-patent periods

C57BL/6 mice were infected with WT GFP or *Pbstk2* KO sporozoites through intravenous injection of sporozoites. Parasitaemia of infected mice was monitored daily by Giemsa-stained blood smear.

### *In vitro* EEF culture and Immunofluorescence assays

Human liver hepatocellular carcinoma (HepG2) cells (5.0×10^4^ per well) were seeded in a 48-well plate, 24 h before prior to addition of sporozoites. The cultures were maintained in DMEM containing 10% FCS as described in the earlier section, in addition to 1× antibiotic and antimycotic. Nearly 5×10^3^ sporozoites of either WT GFP or *Pbstk2* KO were added per well and centrifuged at 320 ***g*** for 4 min. To quantify EEF development, sporozoites were added to HepG2 cells and maintained for different time points and fixed with 4% paraformaldehyde. The EEFs in different stages of development were stained with 1:1000 dilution of anti-UIS4 antibody (Mueller et al., 2005a) specific for the parasitophorous vacuolar membrane (PVM) and revealed with 1:300 dilution of anti-rabbit Alexa Fluor 594 antibody (Thermo Fisher Scientific, Cat No. A-11012). To stain hepatic merozoites, a monoclonal antibody 25.1 specific for merozoite surface protein 1 (MSP1) was used at 1:5000 dilution (Holder and Freeman, 1981), and the immunoreactivity was revealed using 1:300 dilution of anti-mouse Alexa Flour 594 (Thermo Fisher Scientific, Cat No. A21203). The host and parasite nuclei were stained with Hoechst 33342 (Thermo Fisher Scientific, Cat No. H1399).

## Supplementary Material

Supplementary information

## References

[BIO042028C1] Al-Nihmi F. M. A., Kolli S. K., Reddy S. R., Mastan B. S., Togiri J., Maruthi M., Gupta R., Sijwali P. S., Mishra S. and Kumar K. A. (2017). A novel and conserved plasmodium sporozoite membrane protein SPELD is required for maturation of exo-erythrocytic forms. *Sci. Rep.* 7, 40407 10.1038/srep40407 28067322 PMC5220379

[BIO042028C2] Bushell E., Gomes A. R., Sanderson T., Anar B., Girling G., Herd C., Metcalf T., Modrzynska K., Schwach F., Martin R. E. et al. (2017). Functional profiling of a plasmodium genome reveals an abundance of essential genes. *Cell* 170, 260-272.e8. 10.1016/j.cell.2017.06.030 28708996 PMC5509546

[BIO042028C3] Falae A., Combe A., Amaladoss A., Carvalho T., Menard R. and Bhanot P. (2010). Role of Plasmodium berghei cGMP-dependent protein kinase in late liver stage development. *J. Biol. Chem.* 285, 3282-3288. 10.1074/jbc.M109.070367 19940133 PMC2823412

[BIO042028C4] Goldsmith E. J., Akella R., Min X., Zhou T. and Humphreys J. M. (2007). Substrate and docking interactions in serine/threonine protein kinases. *Chem. Rev.* 107, 5065-5081. 10.1021/cr068221w 17949044 PMC4012561

[BIO042028C5] Holder A. A. and Freeman R. R. (1981). Immunization against blood-stage rodent malaria using purified parasite antigens. *Nature* 294, 361-364. 10.1038/294361a0 7312033

[BIO042028C6] Hopp C. S., Flueck C., Solyakov L., Tobin A. and Baker D. A. (2012). Spatiotemporal and functional characterisation of the Plasmodium falciparum cGMP-dependent protein kinase. *PLoS ONE* 7, e48206 10.1371/journal.pone.0048206 23139764 PMC3489689

[BIO042028C7] Janse C. J., Ramesar J. and Waters A. P. (2006). High-efficiency transfection and drug selection of genetically transformed blood stages of the rodent malaria parasite Plasmodium berghei. *Nat. Protoc.* 1, 346-356. 10.1038/nprot.2006.53 17406255

[BIO042028C8] Kappe S. H., Buscaglia C. A., Bergman L. W., Coppens I. and Nussenzweig V. (2004). Apicomplexan gliding motility and host cell invasion: overhauling the motor model. *Trends Parasitol.* 20, 13-16. 10.1016/j.pt.2003.10.011 14700584

[BIO042028C9] Kuang D., Qiao J., Li Z., Wang W., Xia H., Jiang L., Dai J., Fang Q. and Dai X. (2017). Tagging to endogenous genes of Plasmodium falciparum using CRISPR/Cas9. *Parasit. Vectors* 10, 595 10.1186/s13071-017-2539-0 29197418 PMC5712073

[BIO042028C10] Kumar K. A., Oliveira G. A., Edelman R., Nardin E. and Nussenzweig V. (2004). Quantitative Plasmodium sporozoite neutralization assay (TSNA). *J. Immunol. Methods* 292, 157-164. 10.1016/j.jim.2004.06.017 15350520

[BIO042028C11] Larkin M. A., Blackshields G., Brown N. P., Chenna R., Mcgettigan P. A., Mcwilliam H., Valentin F., Wallace I. M., Wilm A., Lopez R. et al. (2007). Clustal W and Clustal X version 2.0. *Bioinformatics* 23, 2947-2948. 10.1093/bioinformatics/btm404 17846036

[BIO042028C12] Lasonder E., Janse C. J., Van Gemert G. J., Mair G. R., Vermunt A. M., Douradinha B. G., Van Noort V., Huynen M. A., Luty A. J., Kroeze H. et al. (2008). Proteomic profiling of Plasmodium sporozoite maturation identifies new proteins essential for parasite development and infectivity. *PLoS Pathog.* 4, e1000195 10.1371/journal.ppat.1000195 18974882 PMC2570797

[BIO042028C13] Letunic I. and Bork P. (2016). Interactive tree of life (iTOL) v3: an online tool for the display and annotation of phylogenetic and other trees. *Nucleic Acids Res.* 44, W242-W245. 10.1093/nar/gkw290 27095192 PMC4987883

[BIO042028C14] Matuschewski K. (2006). Getting infectious: formation and maturation of Plasmodium sporozoites in the Anopheles vector. *Cell. Microbiol.* 8, 1547-1556. 10.1111/j.1462-5822.2006.00778.x 16984410

[BIO042028C15] Mcnamara C. W., Lee M. C., Lim C. S., Lim S. H., Roland J., Simon O., Yeung B. K., Chatterjee A. K., Mccormack S. L., Manary M. J. et al. (2013). Targeting plasmodium PI(4)K to eliminate malaria. *Nature* 504, 248-253. 10.1038/nature12782 24284631 PMC3940870

[BIO042028C16] Mota M. M., Pradel G., Vanderberg J. P., Hafalla J. C., Frevert U., Nussenzweig R. S., Nussenzweig V. and Rodriguez A. (2001). Migration of Plasmodium sporozoites through cells before infection. *Science* 291, 141-144. 10.1126/science.291.5501.141 11141568

[BIO042028C17] Mueller A. K., Camargo N., Kaiser K., Andorfer C., Frevert U., Matuschewski K. and Kappe S. H. (2005a). Plasmodium liver stage developmental arrest by depletion of a protein at the parasite-host interface. *Proc. Natl. Acad. Sci. USA* 102, 3022-3027. 10.1073/pnas.0408442102 15699336 PMC548321

[BIO042028C18] Okonechnikov K., Golosova O., Fursov M. and Team U. (2012). Unipro UGENE: a unified bioinformatics toolkit. *Bioinformatics* 28, 1166-1167. 10.1093/bioinformatics/bts091 22368248

[BIO042028C19] Osawa M., Tokumitsu H., Swindells M. B., Kurihara H., Orita M., Shibanuma T., Furuya T. and Ikura M. (1999). A novel target recognition revealed by calmodulin in complex with Ca2+-calmodulin-dependent kinase kinase. *Nat. Struct. Biol.* 6, 819-824. 10.1038/12271 10467092

[BIO042028C20] Panchal D. and Bhanot P. (2010). Activity of a trisubstituted pyrrole in inhibiting sporozoite invasion and blocking malaria infection. *Antimicrob. Agents Chemother.* 54, 4269-4274. 10.1128/AAC.00420-10 20643897 PMC2944608

[BIO042028C21] Pereira S. F., Goss L. and Dworkin J. (2011). Eukaryote-like serine/threonine kinases and phosphatases in bacteria. *Microbiol. Mol. Biol. Rev.* 75, 192-212. 10.1128/MMBR.00042-10 21372323 PMC3063355

[BIO042028C22] Prudencio M., Rodriguez A. and Mota M. M. (2006). The silent path to thousands of merozoites: the Plasmodium liver stage. *Nat. Rev. Microbiol.* 4, 849-856. 10.1038/nrmicro1529 17041632

[BIO042028C23] Renia L., Miltgen F., Charoenvit Y., Ponnudurai T., Verhave J. P., Collins W. E. and Mazier D. (1988). Malaria sporozoite penetration. A new approach by double staining. *J. Immunol. Methods* 112, 201-205. 10.1016/0022-1759(88)90358-4 3047262

[BIO042028C24] Silva-Neto M. A., Atella G. C. and Shahabuddin M. (2002). Inhibition of Ca2+/calmodulin-dependent protein kinase blocks morphological differentiation of plasmodium gallinaceum zygotes to ookinetes. *J. Biol. Chem.* 277, 14085-14091. 10.1074/jbc.M107903200 11827960

[BIO042028C25] Sinden R. E., Hartley R. H. and Winger L. (1985). The development of Plasmodium ookinetes in vitro: an ultrastructural study including a description of meiotic division. *Parasitology* 91, 227-244. 10.1017/S0031182000057334 3906519

[BIO042028C26] Sinnis P. and Zavala F. (2008). The skin stage of malaria infection: biology and relevance to the malaria vaccine effort. *Future Microbiol.* 3, 275-278. 10.2217/17460913.3.3.275 18505393

[BIO042028C27] Solyakov L., Halbert J., Alam M. M., Semblat J. P., Dorin-Semblat D., Reininger L., Bottrill A. R., Mistry S., Abdi A., Fennell C. et al. (2011). Global kinomic and phospho-proteomic analyses of the human malaria parasite Plasmodium falciparum. *Nat. Commun.* 2, 565 10.1038/ncomms1558 22127061

[BIO042028C28] Stewart M. J. and Vanderberg J. P. (1988). Malaria sporozoites leave behind trails of circumsporozoite protein during gliding motility. *J. Protozool.* 35, 389-393. 10.1111/j.1550-7408.1988.tb04115.x 3054075

[BIO042028C29] Sturm A., Amino R., Van De Sand C., Regen T., Retzlaff S., Rennenberg A., Krueger A., Pollok J. M., Menard R. and Heussler V. T. (2006). Manipulation of host hepatocytes by the malaria parasite for delivery into liver sinusoids. *Science* 313, 1287-1290. 10.1126/science.1129720 16888102

[BIO042028C30] Sur S. and Agrawal D. K. (2016). Phosphatases and kinases regulating CDC25 activity in the cell cycle: clinical implications of CDC25 overexpression and potential treatment strategies. *Mol. Cell. Biochem.* 416, 33-46. 10.1007/s11010-016-2693-2 27038604 PMC4862931

[BIO042028C31] Szklarczyk D., Franceschini A., Wyder S., Forslund K., Heller D., Huerta-Cepas J., Simonovic M., Roth A., Santos A., Tsafou K. P. et al. (2015). STRING v10: protein-protein interaction networks, integrated over the tree of life. *Nucleic Acids Res.* 43, D447-D452. 10.1093/nar/gku1003 25352553 PMC4383874

[BIO042028C32] Tewari R., Straschil U., Bateman A., Böhme U., Cherevach I., Gong P., Pain A. and Billker O. (2010). The systematic functional analysis of Plasmodium protein kinases identifies essential regulators of mosquito transmission. *Cell Host Microbe* 8, 377-387. 10.1016/j.chom.2010.09.006 20951971 PMC2977076

[BIO042028C33] Vaughan A. M., O'neill M. T., Tarun A. S., Camargo N., Phuong T. M., Aly A. S., Cowman A. F. and Kappe S. H. (2009). Type II fatty acid synthesis is essential only for malaria parasite late liver stage development. *Cell. Microbiol.* 11, 506-520. 10.1111/j.1462-5822.2008.01270.x 19068099 PMC2688669

[BIO042028C34] Ward P., Equinet L., Packer J. and Doerig C. (2004). Protein kinases of the human malaria parasite Plasmodium falciparum: the kinome of a divergent eukaryote. *BMC Genomics* 5, 79 10.1186/1471-2164-5-79 15479470 PMC526369

[BIO042028C35] WHO (2017). *World malaria report 2017*, Geneva, Switzerland.

[BIO042028C36] Wierk J. K., Langbehn A., Kamper M., Richter S., Burda P. C., Heussler V. T. and Deschermeier C. (2013). Plasmodium berghei MAPK1 displays differential and dynamic subcellular localizations during liver stage development. *PLoS ONE* 8, e59755 10.1371/journal.pone.0059755 23544094 PMC3609774

[BIO042028C37] Yoshida N., Nussenzweig R. S., Potocnjak P., Nussenzweig V. and Aikawa M. (1980). Hybridoma produces protective antibodies directed against the sporozoite stage of malaria parasite. *Science* 207, 71-73. 10.1126/science.6985745 6985745

[BIO042028C38] Zhang M., Wang C., Otto T. D., Oberstaller J., Liao X., Adapa S. R., Udenze K., Bronner I. F., Casandra D., Mayho M. et al. (2018). Uncovering the essential genes of the human malaria parasite Plasmodium falciparum by saturation mutagenesis. *Science* 360, eaap7847 10.1126/science.aap7847 29724925 PMC6360947

