# Supplementary material for: A Plasmodium berghei putative serine-threonine kinase 2 (PBANKA_0311400) is required for late liver stage development and timely initiation of blood stage infection
Source: Biol Open. 2019 Aug 15;8(8):bio042028. doi: 10.1242/bio.042028 (PMC6737972; doi:10.1242/bio.042028)
Supplement: Supplementary information [file biolopen-8-042028-s1.pdf]

Biology Open (2019): doi:10.1242/bio.042028: Supplementary information

Fig S1

A

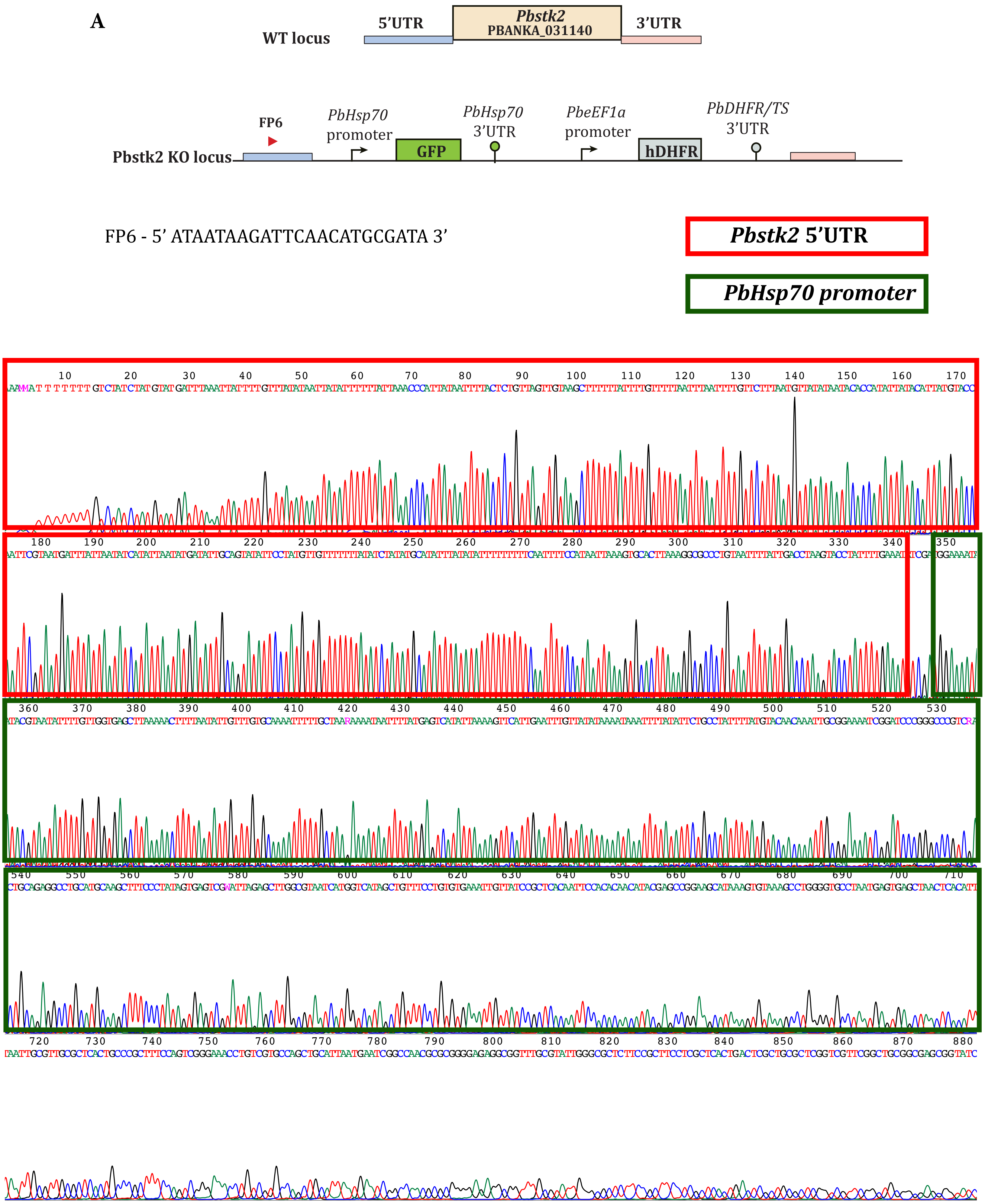

Biology Open • Supplementary information

Biology Open (2019): doi:10.1242/bio.042028: Supplementary information

B

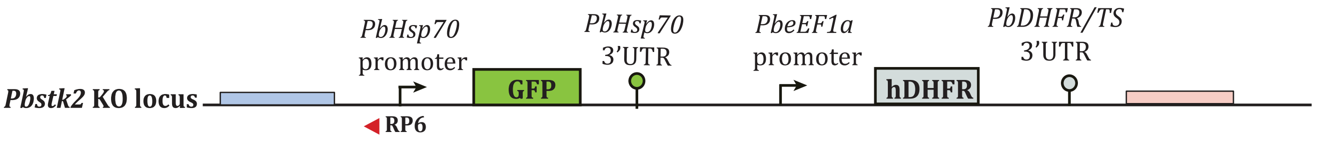

RP6 - TTCCGCAATTTGTTGTACATA

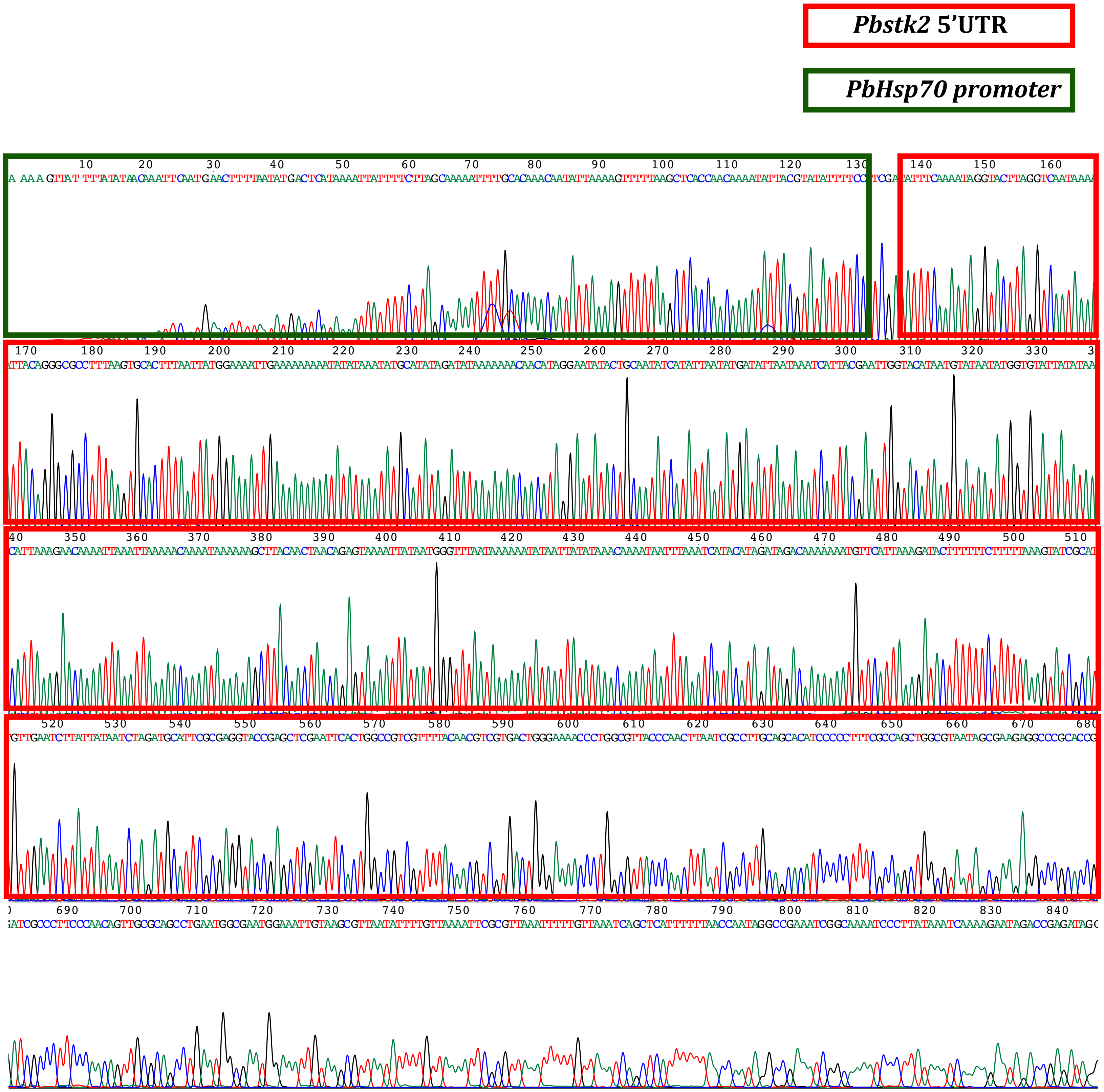

Biology Open • Supplementary information

C

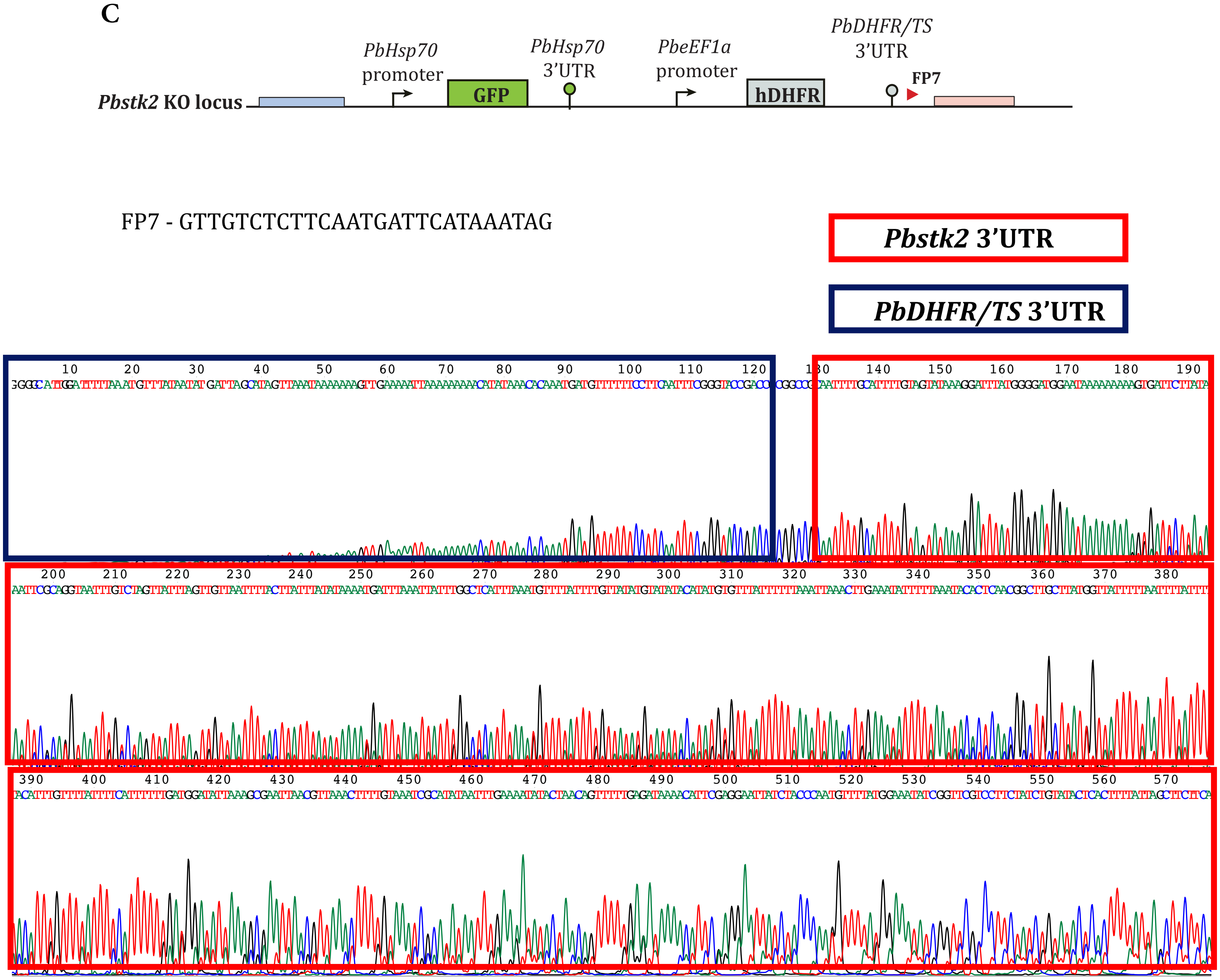

Biology Open • Supplementary information

Biology Open (2019): doi:10.1242/bio.042028: Supplementary information

D

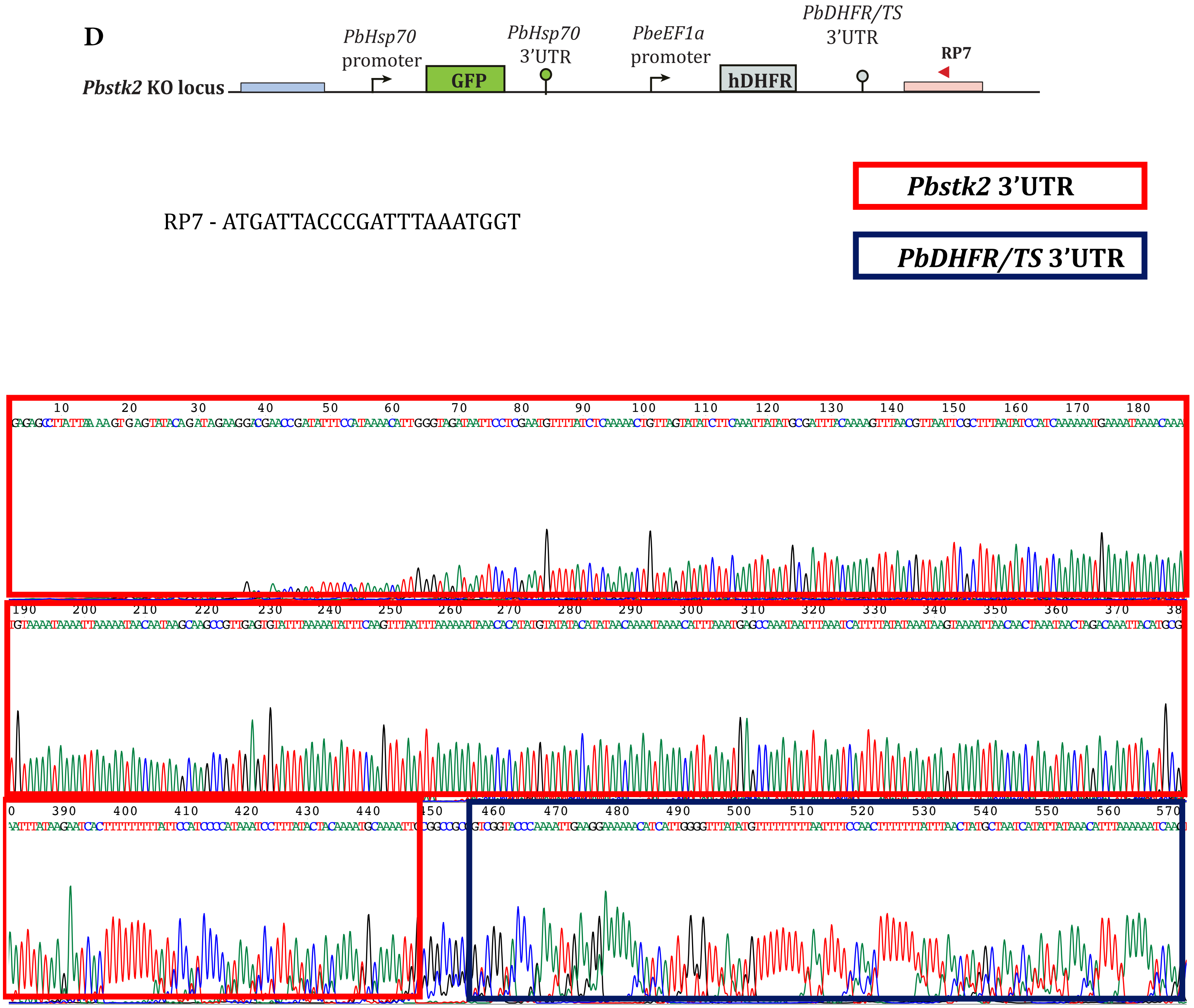

Fig S1

Sequencing of *Pbstk2* KO locus.

A) Forward sequence read using primer FP6 designed in the region of *Pbstk2* 5'UTR
B) Reverse sequence read using RP6 primer designed in the PbHSP70 promoter region
C) Forward sequence read using primer FP7 designed in the region of *PbDHFR/TS* 3' UTR
D) Reverse sequence read using RP7 primer designed in the region of *Pbstk2* 3'UTR

A and B: represent correct integration at 5' region of *Pbstk2* KO locus
C and D: represent correct integration at 3' region of *Pbstk2* KO locus

Biology Open • Supplementary information
